# Contrast-enhanced ultrasound diagnosis and efficacy prediction of primary breast lymphoma: a case report and literature review

**DOI:** 10.3389/fonc.2025.1602270

**Published:** 2025-10-14

**Authors:** Siqi Tang, Yuqin Ma, Xiaochen Zhan, Jianghao Lu, Tingting Wu, Peng Zhou

**Affiliations:** ^1^ Department of Ultrasound, The First Affiliated Hospital of Shenzhen University, The Second People’s Hospital of Shenzhen, Shenzhen, China; ^2^ Department of Pathology, The First Affiliated Hospital of Shenzhen University Health Science Center, Shenzhen Second People’s Hospital, Shenzhen, China

**Keywords:** primary breast lymphoma, ultrasound, contrast-enhanced ultrasound, imaging diagnosis, case report

## Abstract

Primary breast lymphoma (PBL) is a rare entity, accounting for less than 1% of all breast malignancies. PBL is often difficult to differentiate from other benign and malignant breast tumors. This case report describes the contrast-enhanced ultrasound (CEUS) diagnosis of PBL in a 34-year-old female patient, initially presenting with a left breast lump. CEUS revealed some distinctive features: diffuse hyperenhancement with the “floating vessel” sign. These findings enabled a timely diagnosis. Histopathological analysis confirmed diffuse large B-cell lymphoma. After more than two years of chemotherapy, CEUS revealed significant lesion shrinkage with well-defined margins alongside delayed arrival time, reduced peak intensity, and a decreased area under the curve, collectively indicating a favorable chemotherapeutic response. Eventually, the patient underwent surgery, and pathological examination confirmed the absence of significant lymphoma cells following chemotherapy. This case underscores the value of CEUS in identifying PBL and assessing chemotherapy efficacy through qualitative characterization and quantitative parameters, thereby demonstrating its dual role in both lesion identification and therapeutic monitoring. It also emphasizes the significance of including lymphoma in the differential diagnosis of breast masses.

## Introduction

Primary breast lymphoma (PBL) is a rare malignancy, representing less than 1% of all breast cancers and approximately 2% of extranodal lymphomas (1, 2). Its clinical and imaging presentation often mimics that of breast carcinoma or benign lesions, posing a significant diagnostic challenge (2, 3). Timely and accurate diagnosis is crucial for effective treatment planning and improved patient outcomes. Imaging modalities such as computed tomography (CT), magnetic resonance imaging (MRI) and conventional ultrasound (CUS) play a pivotal role in the diagnostic workup of breast lymphoma.

CUS is the most commonly used imaging modality for breast lesions due to its accessibility, affordability, and non-invasiveness (2, 4). CUS typically reveal a hypoechoic mass with posterior acoustic enhancement and lack of microcalcifications, yet these findings overlap significantly with other breast pathologies (5). Contrast-enhanced ultrasound (CEUS), as an advanced ultrasound technique, has unique advantages in displaying micro-vessels and enhances the visualization of blood flow within tissues, providing additional diagnostic information (6, 7). Studies suggest that CEUS features of Lymphomas—such as marked homogeneous hyperenhancement and the “floating vessel” sign—may help distinguish it from other tumors (8). The “floating vessel” sign, which indicates a large vessel encased by a mass without vascular involvement. Furthermore, emerging evidence indicates that quantitative CEUS parameters—such as arrival time (AT), peak intensity (PI), time to peak (TTP), and area under the curve (AUC)—may not only aid in diagnosis but also hold potential for predicting and monitoring treatment response in lymphomas treated with regimens such as R-CHOP (4, 9–11).

This case report focuses on a rare instance of PBL diagnosed and efficacy predicted through CEUS and discusses the diagnostic challenges and the utility of this new technology.

## Case presentation

### Clinical presentation

A 34-year-old female patient was admitted to the Department of Thyroid and Breast Surgery on July 10, 2021, following the incidental self-detection of an egg-sized, painless lump in her left breast one month prior. She denied any associated symptoms such as pain, redness, fever, chest tightness, cough, expectoration, nausea, or vomiting. The patient had no personal history of malignant tumors. A detailed timeline of her diagnostic and therapeutic course is summarized in **Table 1**.

**Table 1:** Diagnostic, therapeutic, and follow-up timeline for the patient with primary breast lymphoma.

Date	Category	Event
July 2021	Symptom Onset	Self-detection of a painless lump in the left breast
10 Jul 2021	Clinical Visit	Initial presentation to the Department of Thyroid and Breast Surgery
12 Jul 2021	Imaging Diagnosis	Multimodal ultrasound (CEUS) and breast MRI performed
14 Jul 2021	Pathological Confirmation	Ultrasound-guided core biopsy confirmed non-Hodgkin B-cell lymphoma
22 Jul 2021	Metabolic Imaging	PET/CT revealed a hypermetabolic breast lesion (SUV max 16.1) and reactive axillary nodes
27 Jul 2021–5 Jan 2022	CNS Prophylaxis	4 sessions of intrathecal chemotherapy (methotrexate/cytarabine/dexamethasone)
30 Jul 2021–Jan 2022	Chemotherapy	7 cycles of R-CHOP regimen completed
17 Mar 2022–Sep 2023	Maintenance Therapy	18 cycles of lenalidomide (25 mg/day, d1–21, q4w)
28 Mar 2024	Preoperative Assessment & Surgery	Preoperative CEUS followed by lumpectomy, histopathology showed no residual lymphoma
Postoperative	Follow-up	Ultrasound at 6 months showed no abnormalities

### Initial CEUS

**Figure 1:**
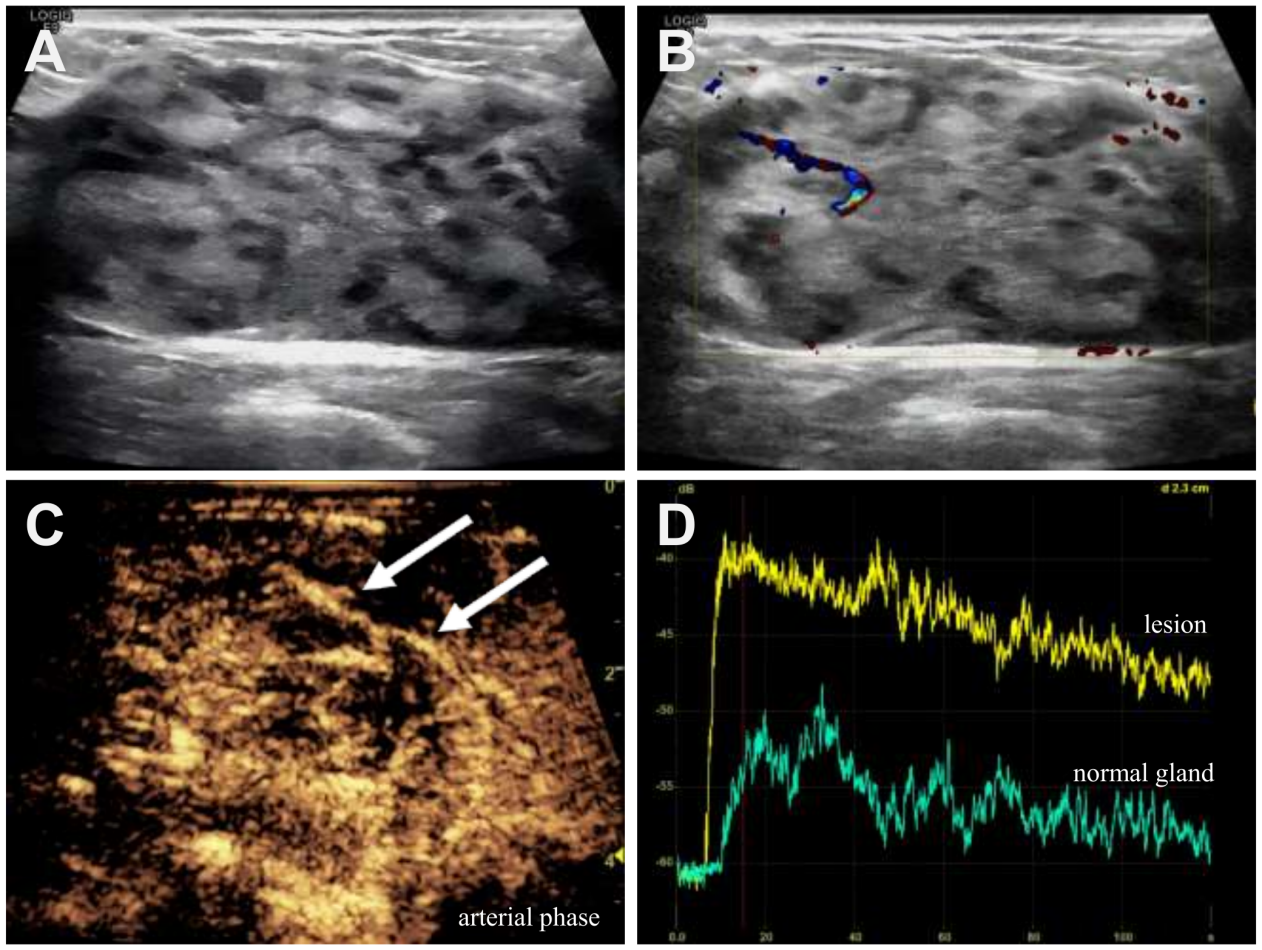
Patient underwent multimodal ultrasound examination for disease diagnosis. **(A)** Gray - scale ultrasonography showed a 57×51×27mm hypoechoic, thickened, disordered area at 3 o’clock in the left breast. It had ill-defined borders, inhomogeneous internal echo, similar to adjacent glandular tissue. **(B)** CDFI detected a blood flow signal in the lesion. **(C)** During the arterial phase, CEUS revealed a “floating vessel” sign (indicated by the arrow) within the lesion, accompanied by homogeneous enhancement of the surrounding tissues. **(D)** Quantitative CEUS time-intensity curve. The yellow and green curves represent the lesion and the surrounding glandular tissue, respectively.

The patient underwent multimodal ultrasound evaluation using a GE LOGIQ E9 system with a linear array transducer (frequency range: 4–15 MHz). The mechanical index (MI) was set at 0.06–0.08 for CEUS. Gray-scale imaging identified a hypoechoic, architecturally distorted area at the 3 o’clock position of the left breast, measuring approximately 57 mm × 51 mm × 27 mm, with echogenicity similar to the surrounding glandular tissue, ill-defined margins, and internal heterogeneity (**Figure 1A**). Color Doppler flow imaging (CDFI) revealed neovascularization within the lesion (**Figure 1B**). Bilateral axillary lymph nodes were enlarged with cortical thickening.

A 4.8 mL bolus of sulfur hexafluoride microbubble contrast agent (SonoVue^®^, Bracco, Milan, Italy) was administered intravenously via the left median cubital vein, followed by a 5 mL saline flush. CEUS was performed in contrast-specific mode with low MI. The lesion showed diffuse hyperenhancement with the presence of “floating vessel” sign –a characteristic observation wherein enhanced vascular structures appear to be suspended or floating within the diffusely enhancing glandular tissue, without clear evidence of vascular stenosis, encirclement, or invasion (**Figure 1C**). Quantitative analysis was performed using the built-in Q-Contrast^®^ software (GE Healthcare). Enhancement parameters included an AT of 7.47 s, PI of –38.80 dB at 18.14 s and AUC (a parameter reflecting total blood volume) of 981.88 dB·s (**Figure 1D**). The lesion exhibited rapid wash-in and delayed wash-out kinetics. The overall imaging features were classified as ACR BI-RADS 4C, suggestive of breast lymphoma.

### Further diagnostic investigations

Breast MRI (**Supplementary Figure 1**) indicated the presence of a mass in the upper-outer and lower-outer quadrants of the left breast, measuring approximately 79.6 mm × 47.1 mm. On T1-weighted imaging, it showed mixed high-and-low signal; on T2-weighted imaging, it presented high signal; on diffusion-weighted imaging, it displayed high signal, and on apparent diffusion coefficient mapping, it showed low signal. The mass was inhomogeneous and demonstrated disordered glandular structure on plain scan, with inhomogeneous enhancement after contrast administration. The time-signal intensity curve was of the plateau type, leading to an ACR BI-RADS category 5 assessment.

**Figure 2:**
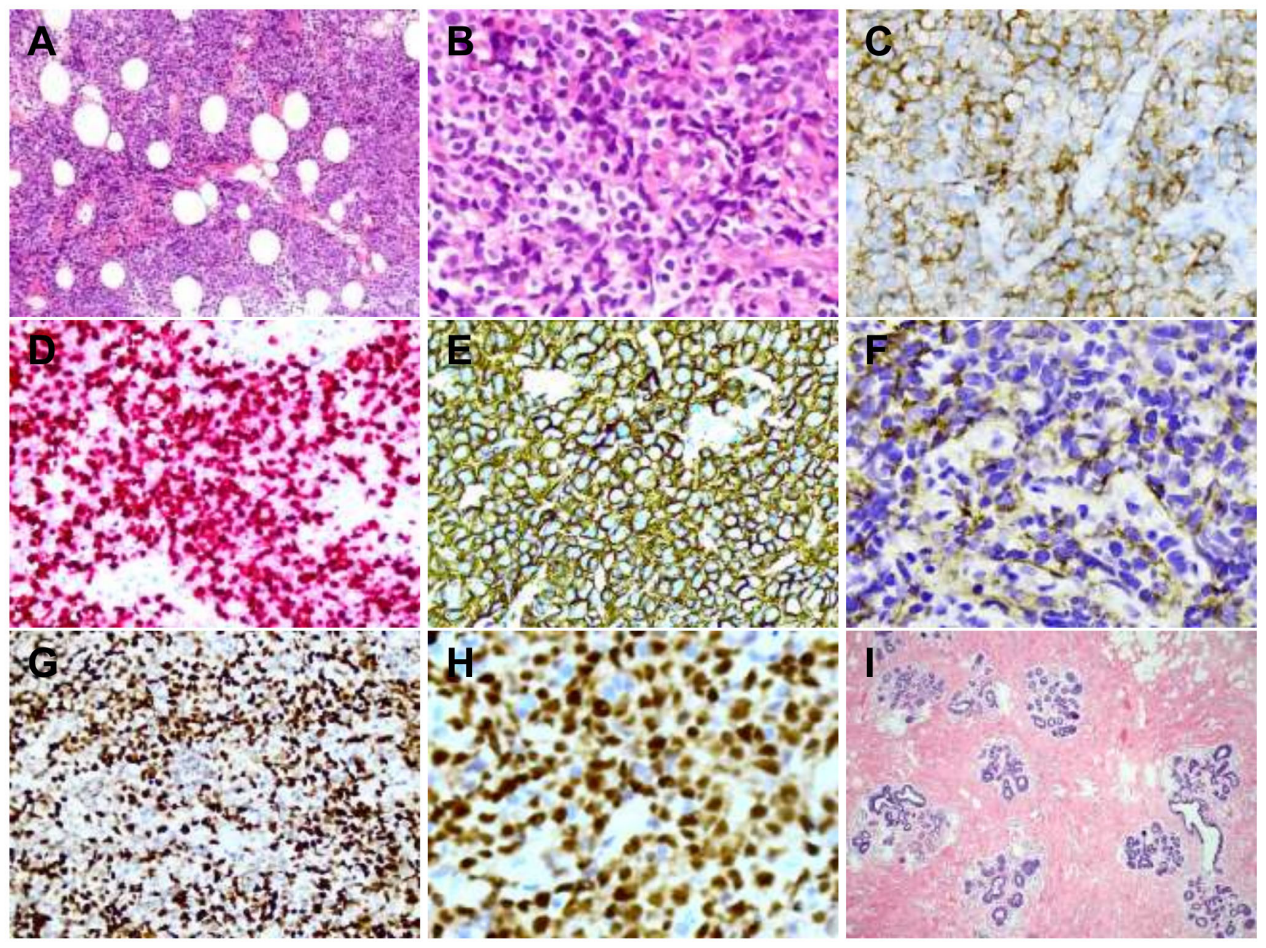
Preoperative and postoperative histological features. Preoperative histology and immunohistochemistry results **(A–H)** HE staining showed tumor cells were diffusely distributed and invaded the adipose tissue from **(A)** (X200) and **(B)** (X400). Immunohistochemical staining revealing CD19(+), Ki - 67(80%+), CD21(+), CD10(weak +), and Bcl- 6(+)/MUM-1(+) expression **(C–H)** (X400). Postoperative histology result **(I)**: HE staining showed adenosis changes (X200).

Laboratory tests showed decreased monocyte percentage and elevated uric acid. On July 14, an ultrasound-guided core needle biopsy using a 16-gauge (G) needle was performed on the left breast mass, obtaining adequate tissue cores for histopathological and immunohistochemical analysis. Pathological and immunohistochemical analyses confirmed diffuse large B-cell lymphoma (DLBCL). Molecular studies revealed negative EBV EBER *in-situ* hybridization, BCL2/MYC gene integrity, BCL6 gene breakage positivity, and wild-type MYD88. Immunohistochemistry results of tumor cells showed AE1/AE3(-), GATA3(-), LCA(+), CD3(-), CD20(+), Ki-67(80%+), CD19(+), CD10(weak +), Bcl - 6(+), MUM - 1(+), CD5(-), Bcl - 2(-), CD30(-), C - MYC(+, 30%), CyclinD1(-), TdT(-), CD23(-), CD43(-) (**Figures 2A-H**). Axillary lymph node biopsy revealed no tumor cells.

On July 22, 2021, an external Positron Emission Tomography-Computed Tomography (PET/CT) scan (images not available) demonstrated intense radiotracer uptake in the upper-outer and lower-outer quadrants of the left breast, showing a mass-like hypermetabolic lesion measuring approximately 59 mm × 48 mm × 58 mm with a maximum standardized uptake value (SUV max) of 16.1. Corresponding CT images revealed a well-defined soft tissue lesion with homogeneous density (CT value approximately 39.0 HU). Additionally, multiple mildly enlarged lymph nodes were observed in the bilateral axillae, the largest measuring 13 mm × 10 mm, with mildly increased metabolism (SUV max 3.0). The background liver SUV max was 2.5, and the mediastinal blood pool SUV max was 1.6. The remaining breast parenchyma appeared dense and symmetric without focal lesions or abnormal uptake. These findings were highly consistent with PBL involvement.

### Treatment

The patient was transferred to the Department of Hematology for further management. Bone marrow aspiration and flow cytometry showed no involvement.

Prior to initiation of chemotherapy, the patient received prephase treatment with oral prednisone 30 mg twice daily starting from July 28, 2021. Subsequently, the patient commenced systemic chemotherapy with the R-CHOP regimen (rituximab, cyclophosphamide, doxorubicin, vincristine, and prednisone) on July 30, 2021, completing a total of 7 cycles (on July 30, August 20, September 11, November 2, November 24, and December 15, 2021). Each 21-day cycle was administered as follows:

Day 0: Intravenous infusion of rituximab 600 mg;
Day 1: Intravenous infusion of cyclophosphamide 1.2 g, doxorubicin 60 mg, and vincristine 2 mg;
Days 1–5: Oral prednisone 100 mg once daily;
Days 6–21: Treatment interruption for recovery, during which blood counts, liver and kidney function, and other relevant parameters were monitored.

In addition, the patient received intrathecal prophylaxis with methotrexate 10 mg, cytarabine 50 mg, and dexamethasone 5 mg in 2 mL normal saline on the following dates: July 27, November 1, November 25, 2021, and January 5, 2022, to prevent central nervous system involvement.

All intravenous drugs were administered in a hospital setting. The treatment plan was tailored based on the patient’s age, performance status, disease stage, and treatment response.

From March 17, 2022, the patient began maintenance therapy with lenalidomide (25 mg orally once daily, days 1–21 of each 28-day cycle) for a total of 18 cycles, which continued until September 2023.

### Follow-up CEUS

**Figure 3:**
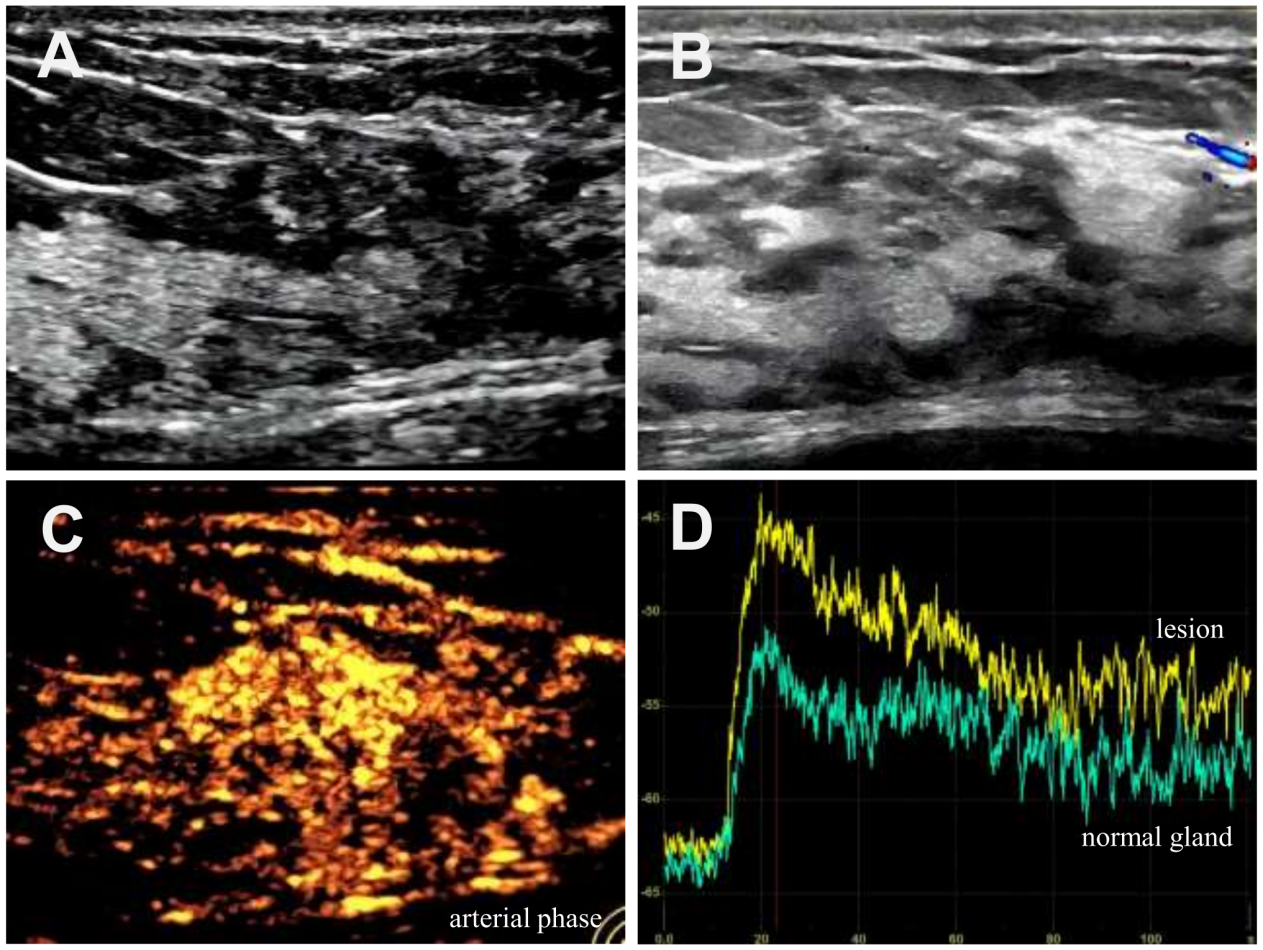
Patient underwent multimodal ultrasound examination after chemotherapy. **(A)** Gray-scale ultrasonography revealed a structurally disordered area, measuring approximately 26×11 mm, at the 3 - o’clock position of the left breast. This area had an irregular shape, inhomogeneous internal echogenicity, and well-defined margins. **(B)** CDFI detected no evident blood flow signals either around or within the nodule. **(C)** On arterial-phase CEUS, the lesion developed a well-defined margin after contrast enhancement. Contrast agent within the lesion was heterogeneous, and no penetrating vessels were detected in the perilesional area. **(D)** Quantitative CEUS time-intensity curve. The yellow and green curves represent the lesion and the surrounding glandular tissue, respectively.

On March 28, 2024, after discontinuation of all treatment for 6 months, follow-up CUS and CEUS were performed. Gray-scale imaging demonstrated a structurally disordered area measuring approximately 26×11 mm at the 3 o’clock position of the left breast, exhibiting an irregular shape, unclear margins, parallel orientation, and heterogeneous echogenicity without posterior acoustic changes (**Figure 3A**). CDFI revealed no significant vascularity within or around the lesion (**Figure 3B**). On CEUS, the lesion demonstrated centripetal hyperenhancement with well-defined margins and a reduced size of approximately 19×9 mm (**Figure 3C**). It showed heterogeneous contrast distribution without non-perfused areas or penetrating vessels, consistent with a faster wash-in and delayed wash-out pattern. Quantitative analysis (**Figure 3D**) revealed an AT of 12.7 s, PI of –45.90 dB, and AUC of 502.88 dB·s. Compared with pre-chemotherapy values (AT: 7.47 s, PI: –38.80 dB, AUC: 981.88 dB·s), the reduced changes in the ratio of PI and AUC were 18.3% and 48.7%, indicate significantly attenuated perfusion, suggesting a favorable treatment response. The inter-observer reproducibility for CEUS parameters (AT, PI, and AUC) in our study was acceptable and satisfactory (ICC > 0.75). These CEUS findings collectively reflect marked reduction in lesion vascularity and size after chemotherapy. Preoperative CEUS was used to localize the lesion prior to excision.

### Surgery

Under CEUS guidance, the patient underwent left breast lumpectomy (local excision of the residual lesion) on March 28, 2024. Lymph node dissection was not performed due to the following reasons: initial biopsy of axillary nodes showed no malignancy, PET-CT indicated only mild metabolic activity suggestive of reactive hyperplasia rather than metastasis, and the primary lymphoma had shown significant response to chemotherapy with no clinical evidence of nodal involvement at the time of surgery. Histopathological examination of the surgical specimen revealed breast adenosis, stromal collagenization, and focal atypical ductal hyperplasia, with no residual lymphoma cells (**Figure 2I**). Examination of all submitted tissue specimens confirmed consistent pathological findings with no evidence of residual tumor. The patient recovered well, and no abnormalities were detected on ultrasound during six months of follow-up.

## Discussion

Breast lymphoma represents an uncommon hematologic malignancy arising from the lymphoid tissue within the breast, encompassing both PBL and secondary breast lymphoma (SBL) (3, 12). The diagnostic criteria for PBL were initially proposed by Wiseman and Liao in 1972 (13). Pathologic specimen showing a close link between mammary tissue and lymphomatous infiltrate, combined with the absence of systemic lymphoma history and specific lymph node involvement patterns, are key determinants in diagnosing PBL (14). SBL represents metastatic involvement of the breast by systemic lymphoma, usually accompanied by disease in other sites (e.g., lymph nodes, spleen) (15).

As a rare manifestation of lymphoma, PBL predominantly originates from B-cell lineages, constituting approximately 50% of all cases. The vast majority of PBL cases are classified as non-Hodgkin lymphoma, with high-grade B-cell lymphomas being the most prevalent (16). Among these, DLBCL is the most frequently observed subtype. Less commonly, PBL may present as follicular lymphoma, mucosa-associated lymphoid tissue lymphoma, or Burkitt lymphoma (2, 3). Notably, breast implant-associated anaplastic large cell lymphoma (BIA-ALCL) is a distinct CD30+ T-cell lymphoma specifically associated with breast implants, classified separately by the WHO (17).

PBL exhibits a strong predilection for females, with the median age of diagnosis typically ranging between 60 and 65 years (14). Clinically, the most common clinical manifestation is a solitary, painless, and palpable mass, observed in approximately 61% of cases, although multifocal lesions may occasionally be present (2). Cutaneous or local inflammatory signs, such as nipple discharge, skin retraction, or erythema, are uncommon and rarely reported (14, 18). Anatomically, PBL demonstrates a slight predominance in the right breast, accounting for 48% of cases, while bilateral involvement is noted in approximately 11% of patients (16).

PBL exhibits imaging characteristics across various modalities, which are crucial in the diagnostic workflow. At mammography, PBL most commonly manifests as a solitary, non-calcified, oval-shaped mass (19). The margins of these masses are typically either circumscribed or indistinct, with spiculated margins being a rare finding. Additionally, global asymmetry is an infrequent presentation of PBL at mammography (20). When evaluated with MRI, PBL masses are isointense on T1WI and hyperintense on T2WI. Enhancement patterns can be either homogeneous or heterogeneous (21). In certain instances, MRI may reveal a spiculated lesion with polycyclic boundaries (22). PET/CT scans of PBL tumors display intense, diffuse hypermetabolism (23). Despite the availability of various imaging modalities mentioned above, CUS and CEUS remain crucial methods for the differential diagnosis of PBL.

In this case, gray-scale ultrasonography detected a thickened and disorganized region with echogenicity similar to adjacent glandular tissue, ill-defined boundaries and inhomogeneous internal echoes. CDFI revealed neovascularization within the lesion. Overall, both grayscale and Doppler sonographic features showed limited specificity (19, 22). Except for homogeneous hyperenhancement and the characteristic “floating vessels” sign, CEUS revealed enhancement features suspicious for malignancy—specifically, a rapid wash-in and delayed wash-out kinetic pattern with a large AUC. Although the CEUS features of PBL have a certain degree of specificity, they are not pathognomonic and must be differentiated from: atypical fibroadenomas and breast carcinoma. The main distinguishing points are summarized in **Supplementary Table 1**. Atypical fibroadenomas are characterized by a regular shape, absence of surrounding vessels penetrating into the lesion, lack of perfusion defects, and no significant change in the enhanced area compared to two-dimensional imaging (24). Breast carcinoma typically shows an enlarged lesion area, heterogeneous perfusion, perfusion defects, and crab-like enhancement (6). Crab-like enhancement refers to irregular, spiculated, or radiating branches of enhancement extending from the margin of the mass into the surrounding breast tissues (6). This reflects the infiltrative growth pattern of carcinomas, which destructively invade adjacent structures and induce chaotic angiogenesis. While, the “floating vessel” sign is own to the lymphoma cells growing diffusely around and preserving the native vascular architecture, rather than destructively invading it (25).There have been no currently published reports on the use of CEUS for diagnosing BIA-ALCL. Therefore, CUS remains the key modality for distinguishing it from PBL for high sensitivity (84%) and specificity (100%) for detecting peri-implant fluid and solid masses in BIA-ALCL (26). Typical findings of BIA-ALCL include a homogeneous peri-implant effusion accompanied by inflammatory changes in surrounding tissue. Masses typically appear as oval, hypoechoic, and well-circumscribed lesions without internal blood flow on CDFI (27).

In this case, we utilized CEUS to dynamically monitor the patient’s lesion changes following chemotherapy. Of particular significance, CEUS serves as a valuable imaging modality not only for the initial diagnosis of breast lymphoma but also for the sequential monitoring and quantitative assessment of chemotherapeutic efficacy (28, 29).The observed lesion regression, coupled with delayed AT, reduced PI, and smaller AUC—parameters indicative of attenuated tumor perfusion dynamics, underscoring the significance of CEUS utility in evaluating chemotherapeutic efficacy for PBL. Our findings are consistent with the majority of current studies. In Lu’s study, CEUS shows promise in assessing non-Hodgkin’s lymphoma response to R-CHOP/CHOP therapy, as perfusion changes precede morphological alterations in indicating treatment efficacy and pretreatment PI values may help predict perfusion response and outcomes (10). However, there are also divergent findings in the existing literature. For instance, a study by Trenker et al. indicated that CEUS does not provide additional predictive value beyond B-mode ultrasound for early prediction of treatment response in lymphoma (30). Therefore, further large-scale studies will be needed to validate its value.

The final diagnosis of PBL relies primarily on core biopsy or surgery results and immunohistochemical staining (31). Compared to core biopsy, surgery can obtain sufficient tissue samples for a more accurate pathological diagnosis. When it comes to the treatment of PBL, the approach remains a subject of debate (32). Although several guidelines have been formulated, there is a lack of unanimity regarding the optimal treatment strategy (33). Treatment usually involves a combination of surgery, radiotherapy, chemotherapy, and immunotherapy (34). The choice of treatment guidelines is contingent upon the histological subtype and stage of the disease (34). For instance, the CHOP regimen has been firmly established as the standard treatment for primary breast DLBCL (33, 34). In this case, following chemotherapy, surgical resection was performed. After the operation, the patient’s condition stayed stable, and breast ultrasounds performed in the six months following the procedure showed no significant abnormalities.

## Conclusion

In conclusion, PBL is very rare and usually manifests as a palpable mass with or without an ipsilateral axillary lymph node and no prior or current history of lymphoma in a middle-aged woman. Although no CEUS characteristics for PBL were reported, we believe when a young woman develops a rapidly growing, large, palpable breast mass, and CEUS reveals floating vessels and homogeneous enhancement of the surrounding lesional tissues, PBL should be highly considered in the differential diagnosis, and a biopsy should be promptly performed. CEUS can provide valuable diagnostic information for PBL. Moreover, the qualitative and quantitative parametric analysis of CEUS provides robust evidence for evaluating chemotherapeutic efficacy in PBL.

## Data Availability

The original contributions presented in the study are included in the article/**Supplementary Material**. Further inquiries can be directed to the corresponding author.
