# Supplementary figures and images for: Contrast-enhanced ultrasound diagnosis and efficacy prediction of primary breast lymphoma: a case report and literature review
Source: Front Oncol. 2025 Oct 14;15:1602270. doi: 10.3389/fonc.2025.1602270 (PMC12558826; doi:10.3389/fonc.2025.1602270)

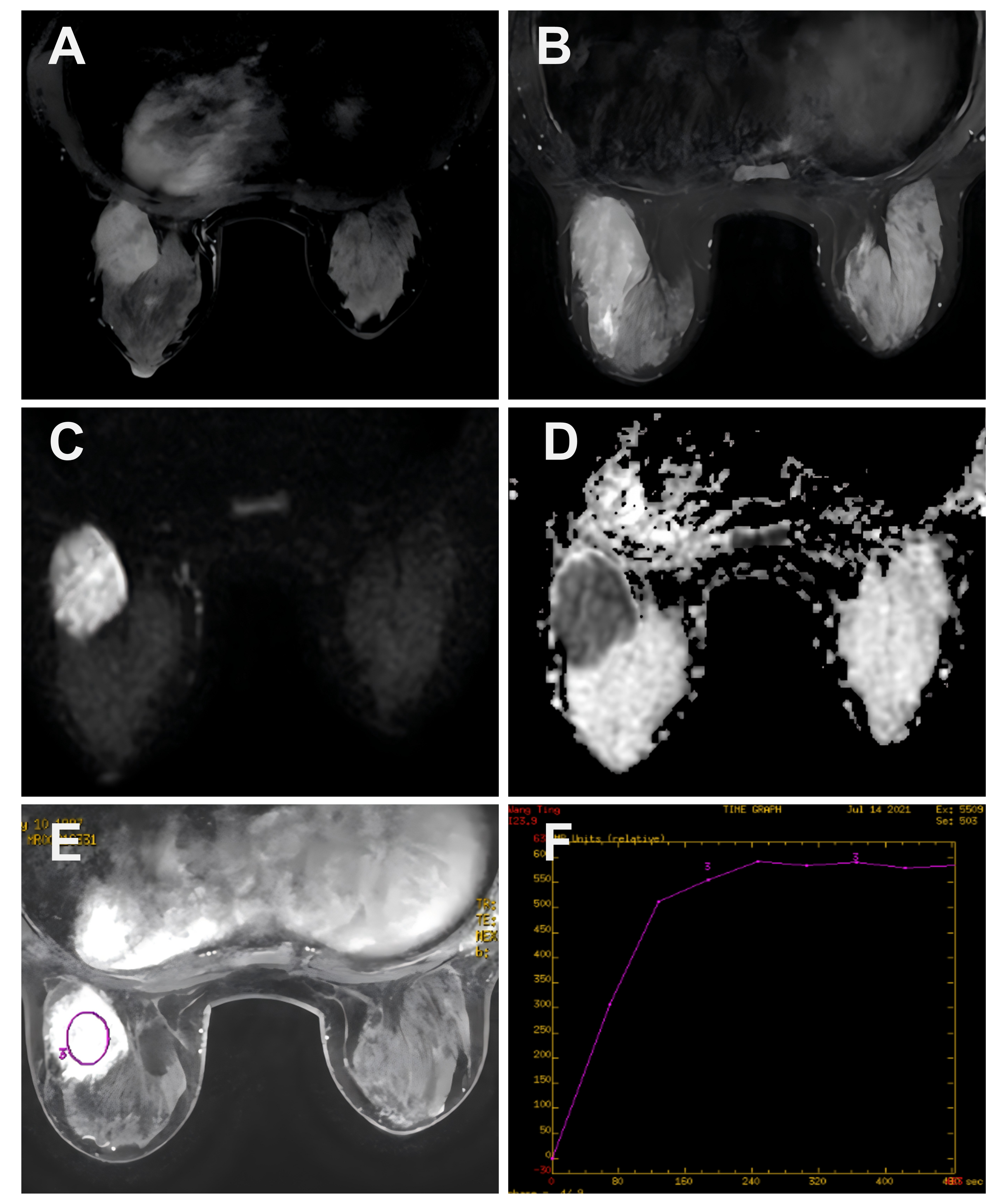

Supplement: Supplementary file 1 [file Image1.tif]
