# Supplementary material for: Contrast-enhanced ultrasound diagnosis and efficacy prediction of primary breast lymphoma: a case report and literature review
Source: Front Oncol. 2025 Oct 14;15:1602270. doi: 10.3389/fonc.2025.1602270 (PMC12558826; doi:10.3389/fonc.2025.1602270)
Supplement: Supplementary file 4 [file Table1.docx]

**Supplementary Table 1. Comparison of CEUS features in PBL, breast carcinoma, and atypical fibroadenoma**

| **CEUS Features** | **PBL** | **Breast Carcinoma** | **Atypical Fibroadenoma** |
| --- | --- | --- | --- |
| Enhanced intensity | hyperenhancement | hyperenhancement | hypoenhancement or  isoenhancement |
| Enhanced boundary | ill-defined | ill-defined | well-defined |
| Enhanced direction | diffuse | centripetal | diffuse or centrifugal |
| Enhancement homogeneity | homogeneous | heterogeneous | homogeneous |
| Enhancement time | rapidly | rapidly | equally or slowly |
| Enhancement scope | enlarged | enlarged | not enlarged |
| Perfusion defects | rare | common | rare |
| Characteristic Sign | Floating vessel | crab-like enhancement | none specific |
